# Fundamental Motor Skills Mediate the Relationship Between Physical Fitness and Soccer-Specific Motor Skills in Young Soccer Players

**DOI:** 10.3389/fphys.2019.00596

**Published:** 2019-05-28

**Authors:** Jakub Kokstejn, Martin Musalek, Pawel Wolanski, Eugenia Murawska-Cialowicz, Petr Stastny

**Affiliations:** ^1^Faculty of Physical Education and Sport, Charles University, Prague, Czechia; ^2^Department of Physiology and Biochemistry, University School of Physical Education, Wrocław, Poland

**Keywords:** pre-pubescence, soccer, skills, talent development, performance, motor control

## Abstract

Fundamental motor skills (FMS) are the basic elements of more complex sport-specific skills and should be mastered at the end of early childhood; however, the relationship between FMS and sport-specific skills has not yet been verified in prepubertal soccer players. Therefore, the aim of this study was to determine the role of FMS in the process of acquiring soccer-specific motor skills (measured using speed dribbling) with regard to physical fitness and biological maturation. Forty male soccer players (11.5 ± 0.3 years of age) at the highest performance level participated in the study. The test of Gross Motor Development – second edition and Unifittest 6–60 were used to assess FMS and physical fitness, respectively. The role of FMS in a complex theoretical model with the relationships between physical fitness, biological maturation and speed dribbling was analyzed by multiple regression path analyses (MRPA). Moderate to strong correlations were found between FMS, physical fitness, and speed dribbling (*r* = 0.56–0.66). Biological maturation did not appear to be a significant predictor of physical fitness or speed dribbling. The MRPA model using FMS as mediator variable between physical fitness and speed dribbling showed a significant indirect effect (standard estimation = −0.31, *p* = 0.001; *R*^2^ = 0.25). However, the direct correlation between physical fitness and speed dribbling was non-significant. Our results showed that FMS significantly strengthened the influence of physical fitness on the performance of speed dribbling, a soccer-specific motor skill, and thus play an important role in the process of acquiring sport-specific motor skills in prepubertal soccer players. When considering the long-term training process, especially during childhood and before puberty, a wide range of FMS activities should be applied for better and possibly faster acquisition of soccer-specific motor skills.

## Introduction

One of the main goals of professional soccer clubs and their youth academies is to develop young, talented players into successful professional players (Huijgen et al., 2013). Many clubs and national associations (e.g., Germany, Belgium, Portugal, Netherlands) have created programs for talent identification and development (TID) to provide the best training environment and conditions for young players with noticeable potential; enrollment in these programs often starts during early adolescence (Deutscher Fuûball Bund, 2009; Huijgen et al., 2014; Leyhr et al., 2018). A player’s success in a soccer match depends on complex multidimensional performance that is influenced by technical, tactical, physical, anthropometric, and mental factors (Reilly et al., 2000; Forsman et al., 2016). To ensure the highest possible efficiency in the TID process, performance tests of different soccer domains outside the context of the soccer match and notational analysis (observation and quantitative/qualitative analysis of the technical and tactical actions performed during a match) are often used in addition to coaches’ expert and highly subjective assessment methods (Aquino et al., 2017).

During the last two decades, technical-tactical skills and physical fitness in particular have been frequently explored and identified as key determinants of young players’ game performance, serving as discriminants between elite, subelite and non-elite youth soccer players (Meylan et al., 2010; Unnithan et al., 2012; Höner et al., 2017; Serrano et al., 2017; Leyhr et al., 2018). In particular, technical skills such as dribbling the ball, passing, shooting and ball mastery are considered critical game rudiments (Rampinini et al., 2009) and have been recognized as important motor factors within TID programs (Vaeyens et al., 2006; Deutscher Fuûball Bund, 2009). Previous research suggests that technical skills develop most rapidly during the prepubertal and pubertal (10–15 years) phases (Vaeyens et al., 2006; Valente-dos-Santos et al., 2012; Leyhr et al., 2018). Specifically, the test of speed dribbling is the best discriminator of performance levels among soccer players (Vaeyens et al., 2006; Huijgen et al., 2009). Elite young players display significantly better performance in strength, speed, agility and aerobic/anaerobic endurance (Vaeyens et al., 2006; Murtagh et al., 2018; Rommers et al., 2018) than subelite young players. However, any connections between these findings and current game performance in youth players should be made with caution because differences in physical fitness and tactical skills are often caused by differences in the speed of biological maturation (Vaeyens et al., 2008; Costa et al., 2010; Meylan et al., 2010; Unnithan et al., 2012; Cumming et al., 2018). Early maturing soccer players generally show higher levels of explosive performance, sprinting, agility and aerobic endurance (Figueiredo et al., 2009; Valente-dos-Santos et al., 2012; Rommers et al., 2018). The relationship between biological maturation and the performance of technical skills is contradictory to the results of some studies confirming the influence of biological maturation status on the performance of technical skills tests (Philippaerts et al., 2006; Rommers et al., 2018) and other studies finding a lack of influence of biological maturation on the performance of technical skills (Figueiredo et al., 2009; Vandendriessche et al., 2012).

Recently, several studies have emphasized the importance of motor coordination, i.e., non-specific motor coordination, in the process of TID in youth soccer (Vandendriessche et al., 2012; Deprez et al., 2014; Deprez D. et al., 2015; Deprez D.N. et al., 2015; Rommers et al., 2018). Furthermore, these studies showed that motor coordination is a significant long-term predictor of specific aerobic fitness and explosive leg power in young soccer players (Deprez et al., 2014; Deprez D. et al., 2015) and does not depend on biological maturation (Vandendriessche et al., 2012; Rommers et al., 2018). However, the direct relationship between motor coordination and specific technical skills (e.g., speed dribbling) was not explored in prepubescent soccer players. In another study, Deprez D.N. et al. (2015) measured motor coordination performance among club players (playing in the two highest youth soccer leagues) and dropout players (those who dropped to lower soccer leagues) over the 8-year period from age 8 years to age 16 years and found that the club players performed significantly better than the dropout players on all motor coordination tasks and on aerobic endurance and speed. The authors suggested that motor coordination performance is essential for discriminating between players in a high-level training program and dropout players from the age of 9 years until late puberty. Although the direct relationship between motor coordination and specific technical skills was not investigated in this study, one could hypothesize that the dropped players had overall worse specific technical skills and worse motor coordination than club players.

In many studies focused on motor development, the term “motor coordination” has been used to denote motor competence, motor proficiency or fundamental motor skills (FMS) to describe goal-directed human movement (Robinson et al., 2015). For the purpose of our study, we decided to use the term FMS to describe the level of general motor competence. In general, according to several key motor development theoretical models, FMS are frequently defined as the “elements” of more advanced complex movements required to participate in sports, games, or other context-specific physical activity (Clark and Metcalf, 2002; Gallahue et al., 2012). However, no clear research evidence indicates whether this theory is valid in prepubertal soccer players. Once FMS are mastered, the learning of sport-specific skills can occur more quickly and be more effective (Gallahue et al., 2012). FMS are traditionally divided into object control/ball/manipulative skills (e.g., throwing, catching, dribbling), locomotor skills (e.g., running, jumping, galloping), and balance/stability skills (e.g., non-locomotor skills such as body rolling, one-foot balance, stretching, twisting) (Gallahue et al., 2012). Although children have the developmental potential to master most FMS by the age of 6 years (Gallahue et al., 2012), recent research highlights that children and adolescent youth do not perform FMS to their expected developmental capabilities (O´Brien et al., 2016). O´Brien et al. (2016) further demonstrated that while levels of FMS vary by country, performance levels remain consistently low, with the majority of children and adolescents failing to surpass 50% mastery in most skills.

To our knowledge, little attention has been paid to the importance of FMS in the process of acquiring technical skills (e.g., dribbling, receiving, or passing a ball) in prepubescent soccer players. Moreover, current research describes the direct relationships between technical skills and other physical, motor control, or morphological factors but have not described how those factors interact with or mediate specific soccer skills. Although there is clear evidence concerning the relationships between FMS, physical fitness and biological maturation, there is a lack of information about the influence of FMS on the performance of soccer technical skills in prepubescent-aged players. We hypothesized that FMS strengthen the influence of physical fitness and biological maturation on technical skills (e.g., speed dribbling the ball). Therefore, the aim of this study was to investigate the role of FMS in the relationships between physical fitness, biological maturation and technical skills in prepubescent soccer players.

## Materials and Methods

### Methodological Approach

Cross-sectional measurement was performed during the competitive part of the soccer season. The participants were familiarized with the experimental protocol 1 week prior to the experiment and did not perform any exhausting activity 72 h before the experiment. After the participants’ body mass (BM) was estimated, they performed the battery of FMS, speed dribbling and physical fitness tests within 1 day (two training sessions). The FMS and sit-ups (part of Unifittest 6–60) tests were performed indoors on a teraflex surface during the morning training session between 9 and 11 am. The rest of physical fitness tests were then conducted during afternoon training session on the outdoor ground with artificial grass between 3 and 5 pm.

### Participants

The research sample consisted of forty U12 soccer players (mean ± SD; age 11.5 ± 0.3 years; height 145 ± 7.0 cm; body mass 37.2 ± 4.1 kg). The players were members of teams from two clubs in the Prague district of the Czechia that participated in the highest Czech youth league level. These two clubs were randomly selected from a basic sample (a total of fourteen clubs in the Prague district) and then were asked to participate in the study. The weekly cycle consisted of four training sessions (7–8 h) focused primarily on technical-tactical skills during exercises and games and one competitive match. The inclusion criteria were a minimum of 6.4 years of experience with organized soccer and full attendance in ongoing habitual training cycles. Exclusion criteria were any medical problems that compromised participation or performance in the study, such as soft tissue injury, delayed muscle soreness, recent illness or recent recovery from injury. The research was approved by the Ethics Committee of the Faculty of Physical Education and Sport, Charles University, and all participants and their parents signed an informed consent form.

### Fundamental Motor Skills

The Bruininks-Oseretsky Test – 2nd edition (BOT-2; short version) was used to assess fundamental fine and gross motor skills (Bruininks, 2005). The BOT-2 has demonstrated high inter-rater reliability (*r* ≥ 0.90), test-retest reliability (*r* ≥ 0.80) and construct validity (Deitz et al., 2007). The short version contains sixteen items divided into eight dimensions (see Table 1). Raw scores from BOT-2 were transformed into standard scores according to age by ASSIST software (MN, United States). Standard scores were then used for the final analysis.

**Table 1:** List of dimensions and items of the BOT-2 motor test.

Fine motor precision	Balance
Drawing lines through crooked paths	Walking forward on a line
Folding paper	Standing on one leg on a balance beam – eyes open
**Fine motor integration**	**Running speed and agility**
Copying a square	One-legged stationary hop
Copying a circle	**Upper limb coordination**
Copying a star	Dropping and catching a ball – both hands
Copying a pencil	Dribbling a ball – alternating hands
**Manual dexterity**	**Strength**
Transferring a penny	Full push-ups
**Bilateral coordination**	Sit-ups – 30 s
Jumping in place – same side synchronized	
Tapping feet and fingers – same side synchronized	

### Physical Fitness Tests

Three physical fitness parameters were measured (shuttle run 4 m × 10 m, standing broad jump, and 20-m progressive shuttle run). These three tests are included in the Unifittest 6–60 test battery, which is standardized for the Czech context (Mekota and Kovar, 1995; Chytrackova, 2002) with a satisfactory level of reliability and validity (Mekota and Kovar, 1995). *Shuttle run* 4 m × 10 m, which assesses coordination and speed, was performed twice by each player, with 3–4 min of rest between the two trials. In a start position, the player stood on the starting line without moving into the space between photocells. The player sprinted to the opposite marker (10 m), turned and returned to the starting line directly adjacent to the photocell gate. This was performed twice to cover a 40-m distance. The time of the faster trial was recorded. An infrared timing gate (Alge Timing GmbH, Lustenau, Austria) placed at approximately hip height was used for the start and finish points. *Standing broad jump*, an indicator of explosive power in the lower limbs, was performed three times by each player, with 2 min of rest between trials. The player stood behind a line marked on the ground. A two-foot takeoff and landing area was used, and players were instructed to jump as far as possible while swinging their arms and bending their knees to provide forward momentum. The longest jump was recorded and used for the analysis. *Progressive shuttle run* 20 m is a measure of maximal aerobic fitness. The player continuously ran between two lines 20 m apart, keeping pace with recorded beeps, which accelerated each minute. The test was stopped when the player failed to reach the line (within two meters) after two consecutive warnings. Finally, from each test item, a standard score was obtained. The composite score of all tests on a scale from 0 to 20 was calculated as a marker of physical fitness.

### Predicted Maturity Offset

Maturity offset was estimated according to Mirwald et al. (2002) equations. Although these equations have been widely used in the sport environment (e.g., Wickel and Eisenmann, 2007; Gastin et al., 2013; Gil et al., 2014; Meyers et al., 2015), studies have pointed to the limits of this predictive method in both sexes (Malina and Kozieł, 2014a,b; Malina et al., 2016). In the current study, we considered the finding of Kozieł and Malina (2018), who stated that maturity offset predicted from the Mirwald equations matched the observed peak high velocity (PHV) in 12-year-old boys, to be important.

Y-PHV = −9.326 + (length of lower limbs ^∗^ sitting height) – (0.001663 ^∗^ [decimal age ^∗^ length of lower limbs]) + (0.007216 ^∗^ [decimal age ^∗^ sitting height]) + 0.02292 ^∗^ [weight/height]

Body weight was assessed with an accuracy of 0.1 kg using medical calibrated weight (CAS DBI-C, Lesak and Zemánek s.r.o., Czechia). A portable anthropometer (A 226, Trystom, spol. s.r.o., Czechia) with a balancing point to determine the right vertical position of the anthropometer was used for the measurement of height and sitting height (0.1 cm).

### Speed Dribbling Test

**FIGURE 1:**
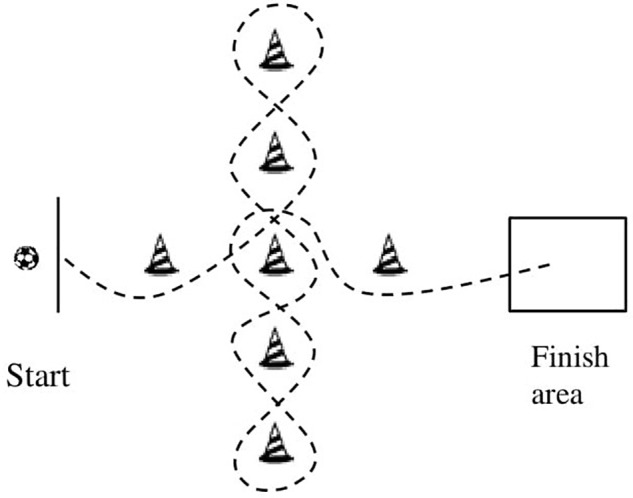
Short dribbling test (Bangsbo and Mohr, 2013).

The short dribbling test (SDT) (Bangsbo and Mohr, 2013; Figure 1) is a test of dribbling the ball at a high speed with changes in direction around a defined track. Players are required to dribble as fast as possible around the cones without touching them. The test is finished by stopping the ball in a square (defined with blue cones) at the end of the track. Each player underwent one training and one competitive trial. If the player touched any cone during dribbling, the trial failed, and the player was allowed an additional trial. Time was measured by a telemetric photocell system (Alge Timing GmbH, Lustenau, Austria), and the best time was recorded. Similar agility dribbling tests have shown acceptable reliability, with an intraclass correlation coefficient between 0.78 and 0.89 and coefficient of variation of 2.4 and 3.9 (Russell et al., 2010; Ali, 2011; Dardouri et al., 2014).

### Data Analysis

Multiple regression path analysis (MRPA) was used to test the hypothesized links, with successive multiple regression equations calculated to estimate path coefficients. Mardia’s, Henze-Zirkler’s and Royston’s multivariate normality tests were performed using the R package MVN, version 4.0.2, in R 3.4.1, with a cut-off of p greater than 0.05 for accepting the normality of multivariate data. The exogenous independent variables were physical fitness and maturity offset. The interacting endogenous variable was FMS. All of these variables were analyzed first using linear regressions and then using multiple regressions. Subsequently, the final path analysis model was selected. In the path model, there were specified direct paths from the exogenous variable to the endogenous variable and from the exogenous and endogenous variables to the SDT. Finally, for variables that had statistically significant predictive power (*p* < 0.05) for FMS or for SDT performance, specific indirect effects via FMS were investigated. MRPA and Pearson’s correlations were performed using M-plus software version 6.0 (Muthen and Muthen, 2010). All data can be found in the “Supplementary Table S1” Supplementary File.

## Results

The means and standard deviations of the basic descriptive statistics and correlation coefficients of FMS, physical fitness, maturity offset and speed dribbling are shown in Tables 2, 3, respectively. Significant moderate associations were observed between speed dribbling and FMS, speed dribbling and physical fitness, and FMS and physical fitness. However, there was no association between speed dribbling and maturity offset.

**Table 2:** Basic descriptive statistics (*n* = 40).

	Mean	SD	Median	Interquartile range	95% confidence interval
Age (years)	11.50	0.30	11.63	0.42	±0.09
Height (cm)	145.00	7.00	149.45	6.53	±1.98
Body mass (kg)	37.20	4.10	37.15	7.85	±2.05
Index BMI (kg/m^2^)	17.52	1.89	17.06	2.08	±0.59
FMS (ss)	57.33	8.88	58.50	17.25	±2.75
Physical fitness (ss)	21.05	3.34	21.00	4.50	±1.03
Maturity offset (years)	−2.88	0.30	−2.90	0.34	±0.09
Speed dribbling (s)	13.68	1.53	13.73	2.46	±0.47

*FMS, fundamental motor skills; SD, standard deviation; ss, standard score.*

**Table 3:** Correlation matrix of the study variables.

	Maturity offset	Physical fitness	FMS
**Maturity offset**	1		
**Physical fitness**	−0.21^∗∗^	1	
**FMS**	−0.29^∗∗^	0.50^∗∗^	1
**Speed dribbling**	−0.03	−0.42^∗∗^	−0.60^∗∗^

*FMS, fundamental motor skills; ^∗∗^indicates statistical significance of p < 0.01.*

In the first step, we analyzed the predictive power of FMS, physical fitness and maturity offset (independent variables) on speed dribbling performance (dependent variable). The linear regression results (Table 4) showed that FMS and physical fitness are significant predictors of speed dribbling performance. Nevertheless, only the effect of FMS (*R*^2^ = 0.36; *t* = 2.97; *p* = 0.003) was significant, while the effect of physical fitness was not (*R*^2^ = 0.18; *t* = 1.64; *p* = 0.100).

**Table 4:** Linear regressions of physical fitness, FMS and maturity offset on speed dribbling.

Independent variable	*B*	*SE B*	β	*t*	*p*-Value
**Physical fitness**	−0.93	0.31	−0.43	3.28	0.001^∗∗^
**FMS**	−3.5	0.74	−0.60	5.95	<0.001^∗∗^
**Maturity offset**	−0.01	0.03	−0.03	0.19	0.85

*FMS, fundamental motor skills; ^∗∗^indicates statistical significance of p < 0.01; B, unstandardized beta; SE B, standard error for the unstandardized beta; β, the standardized beta; t, t-test statistic.*

Since clear evidence of the relationship between biological age and physical fitness in pubescent soccer players has been reported in previous research (biologically advanced players achieve better performance in physical fitness), we verified whether FMS and maturity offset are significant predictors of the level of physical fitness. The multiple regression model showed that the effects of FMS and maturity offset explain 22% of physical fitness performance variability (*R*^2^ = 0.22). Furthermore, from Table 5, it is clear that FMS are significantly better predictors for physical fitness than maturity offset in prepubescent players.

**Table 5:** Multiple regression of FMS and maturity offset on physical fitness.

Independent variable	*B*	*SE B*	β	*t*	*p*-Value
**FMS**	−0.82	0.06	0.48	3.25	0.003^∗∗^
**Maturity offset**	0.18	1.64	0.08	0.50	0.62
**Adjusted *R*^2^**	0.22				

*FMS, fundamental motor skills; ^∗∗^indicates statistical significance of p < 0.01; B, unstandardized beta; SE B, standard error for the unstandardized beta; β, the standardized beta; t, t-test statistic.*

**FIGURE 2:**
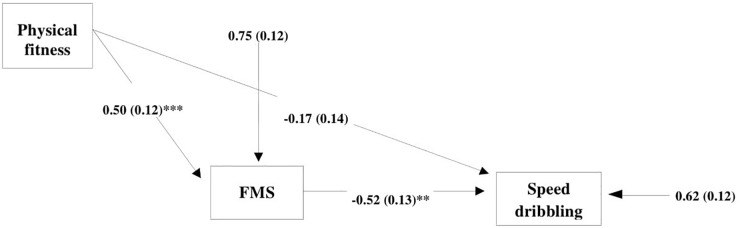
First path analysis model with direct and indirect effects of physical fitness and FMS on speed dribbling [Satorra–Bentler χ^2^ (*df* = 0) = 0; *p* = 0.00; RMSEA = 0.0; SRMR = 0.0; CFI = 0.0; TLI = 0.0]; FMS, fundamental motor skills. ^∗∗^*p* < 0.01; ^∗∗∗^*p* < 0.001.

**FIGURE 3:**
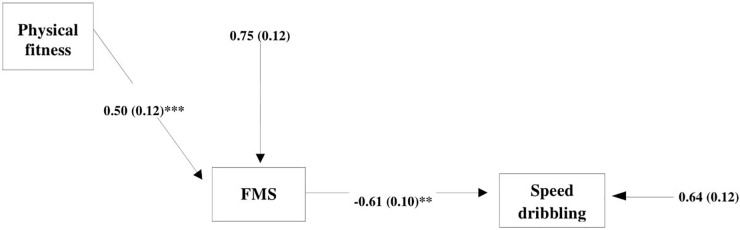
Second path analysis model with only the indirect effect of physical fitness on speed dribbling with FMS as the mediator variable [Satorra–Bentler χ^2^ (*df* = 1) = 1.3; *p* = 0.254; RMSEA = 0.08; SRMR = 0.04; CFI = 0.99; TLI = 0.97]; FMS, fundamental motor skills. ^∗∗^*p* < 0.01; ^∗∗∗^*p* < 0.001.

Considering these findings, we decided to use the analysis model where FMS plays the role of mediator between physical fitness (as the independent variable) and speed dribbling (as the dependent variable representing specific soccer skills). In the 1st path analysis model, we specified both direct and indirect paths between physical fitness and speed dribbling. The 1st path analysis model (Figure 2) showed that the direct effect of physical fitness on speed dribbling was non-significant (standard estimation = −0.17, *p* = 0.247), and the indirect effect through FMS was significant (standard estimation = −0.26, *p* = 0.005). To obtain better results, we decided to formulate the 2nd path analysis model without a direct effect (Figure 3). The 2nd model was approved as significant and acceptable with empirical data explaining more than 25% of the model. Generally, FMS were a significant mediator between physical fitness and speed dribbling (standard estimation = −0.31, *p* = 0.001).

## Discussion

The present study examined the possible role of FMS in the relationships between physical fitness and biological maturation and speed dribbling as a soccer-specific soccer skill in young soccer players. We found that FMS were a significant mediator of the relationship between physical fitness and speed dribbling. Notably, biological maturation did not prove to be a significant contributor to speed dribbling performance through FMS. Despite moderate correlations between physical fitness and speed dribbling, the path model did not reveal a direct influence of physical fitness or biological maturation on speed dribbling. These findings suggest the need for a certain level of FMS (fine and gross motor skills) to acquire soccer-specific motor skills. Generally, both quantitative (physical fitness) and qualitative (FMS) motor aspects were found to be significant contributors to the performance of soccer-specific motor skills, represented by speed dribbling. Our path model revealed that FMS, physical fitness and other related factors have a prior effect on specific skill, whereas biological maturation might explain only 8.7% of motor coordination (Freitas et al., 2016).

Our results suggest that FMS mastery significantly increases the influence of physical fitness on the performance of soccer-specific skills in young players. These findings are in accordance with Clark and Metcalf’s (2002) statement that FMS are basic elements for later skillfulness in a range of sport and game domains. Our results cannot be compared with similar data measured on soccer players. However, similar conclusions have been found in research involving combat sports (Bozanic and Beslija, 2010), where high correlations were found between specific karate skills and FMS (*r* = 0.74) in 5- to 7-year-old members of karate clubs. This study suggested that children with higher FMS also have better karate techniques, while others have difficulties acquiring these techniques. Unfortunately, recent research has documented very poor or insufficient FMS performance in preschool and school-aged children (Okely et al., 2004; Erwin and Castelli, 2008; Hardy et al., 2010; Kokstejn et al., 2017a,b) combined with generally unresolved inactivity in children (Faigenbaum et al., 2018), which may result in impaired acquisition of more complex and difficult sport skills or delays in mastering the required skills. Similarly, the players in our study showed only an average level of FMS even though they were considered to be capable of high performance. Since a higher level of FMS and soccer-specific skills were found in players selected for Belgian professional clubs (Deprez D.N. et al., 2015) than in “dropout” players, FMS and soccer-specific skills seem to be crucial to the identification of gifted players and their likelihood of remaining in high-level talent development programs.

Our participants were at a specific age (U12) where physical development plateaus (in reactive strength and jumping) with a change in the mechanical properties of the lower limb (decreased relative leg stiffness), which has been previously observed (Lloyd et al., 2011). This might explain why biological maturation in U12 children is not strongly related to physical fitness or motor control testing (Freitas et al., 2016) and why separate values of physical fitness and biological maturation are insufficient for predicting a player’s ability to acquire soccer-specific skills. Our path models suggest that the best performance of soccer-specific skills will occur in players with adequate levels of both FMS and physical fitness. This finding is in agreement with the finding that FMS were a long-term predictor of explosive power in soccer players from childhood to young adulthood (Deprez D. et al., 2015). Therefore, we believe that well-developed FMS and the simultaneous development of PF are necessary in pre-PHV boys and that well-coordinated players will improve in power and performance with age due to increased tendon stiffness in late adolescence (Deprez D. et al., 2015).

The harmony between physical fitness and biological maturation has been highlighted by several authors (e.g., Meylan et al., 2010; Unnithan et al., 2012; Vandendriessche et al., 2012) to discriminate elite, subelite and non-elite soccer players during talent identification. Moreover, FMS were found to be a long-term predictor of soccer-specific aerobic performance in elite pubertal soccer players (Deprez et al., 2014) and children (Hands, 2008). Specifically, children with low FMS performed worse on all fitness tests (50-m run, standing broad jump and endurance shuttle run), where endurance shuttle test differences increased between low- and high-FMS groups over 5 years (Hands, 2008). Therefore, we highlight the importance of FMS not only for soccer-specific motor skills but also for separate components of physical fitness, such as explosive power and aerobic endurance, during long-term motor development.

Several possible limitations associated with this study should be noted. The present study utilized a cross-sectional design; thus, the role of FMS in the relationships between physical fitness, biological maturation and soccer-specific motor skills should be interpreted with caution. A longitudinal follow-up of young soccer players, especially during the pubertal phase (aged 12–15 years), may provide a more accurate explanation of this mediation effect. Another possible limitation is related to the non-inclusion of psychological variables such as motivation or self-confidence, which certainly influence game performance and likely also affect the development of new soccer-specific motor skills. Lastly, although several authors consider speed dribbling the most valid soccer skill test, the inclusion of additional soccer-specific skills (e.g., passing, shooting, receiving,or multifaceted tests) could elucidate the role of FMS in the acquisition of soccer-specific motor skills (Vanderford et al., 2004). Therefore, future research should focus on (1) performing longitudinal research to verify the role of FMS in acquiring soccer-specific skills during the pubertal phase, (2) testing more soccer-specific skills and psychological variables with respect to players’ positions, and (3) using a process-oriented (assessment of movement quality) test for FMS assessment.

## Conclusion

This is the first study to evaluate the role of FMS in a complex theoretical model with the relationships between physical fitness, biological maturation and soccer-specific motor skill (measured using speed dribbling) in young soccer players. Our results showed that FMS significantly strengthened the influence of physical fitness on the performance of speed dribbling, a soccer-specific motor skill, and thus play an important role in the process of the acquisition of sport-specific motor skills in prepubertal elite soccer players. Conversely, physical fitness and biological maturation alone did not significantly influence speed dribbling performance. Generally, it appears that developing and improving a wide range of basic FMS as building blocks for more complex and more difficult soccer-specific motor skills is necessary during the long-term training process. Based on these findings, FMS could be included in TID programs for young elite soccer players, especially during childhood and before puberty. Thus, it is recommended that youth soccer coaches and practitioners carefully consider providing training on FMS (fine motor, locomotor, object control, balance), especially during childhood, with an emphasis on the quality of movements.

## Data Availability

All datasets generated for this study are included in the manuscript and/or the Supplementary Files.

## Ethics Statement

This study was carried out in accordance with the recommendations of “name of guidelines, name of committee” with written informed consent from all subjects. All subjects gave written informed consent in accordance with the Declaration of Helsinki. The protocol was approved by the “name of committee.”

## Author Contributions

JK, MM, and PS involved in the conceptualization of the study design and the drafting of the manuscript. JK and MM involved in data collection. JK involved in performing an overview of the previous research. MM involved in conducting the statistical analysis. PW and EM-C helped with the data assessment and interpretation.

## Conflict of Interest Statement

The authors declare that the research was conducted in the absence of any commercial or financial relationships that could be construed as a potential conflict of interest.
