# Supplementary material for: Fundamental Motor Skills Mediate the Relationship Between Physical Fitness and Soccer-Specific Motor Skills in Young Soccer Players
Source: Front Physiol. 2019 May 28;10:596. doi: 10.3389/fphys.2019.00596 (PMC6546828; doi:10.3389/fphys.2019.00596)
Supplement: Supplementary file 1 [file Data_Sheet_1.pdf]

Suplementary Table 1 Values of the study variables in forty male soccer players.

Physical fitness	Maturity Offset	FMS	Speed dribbling
24	-2,86488	64	12,19
24	-3,264625	70	10,95
24	-3,166268	63	11,68
26	-2,758025	67	11,93
27	-3,02691	70	11,81
22	-3,143975	66	12,36
20	-2,642437	59	11,99
22	-2,849132	59	15,21
27	-2,947868	66	12,79
19	-2,254609	70	11,43
15	-2,439063	52	14,42
23	-3,686818	70	12,79
21	-3,042033	67	15,43
18	-2,589528	63	12,45
22	-2,115641	59	12,13
25	-2,642728	54	12,89
27	-2,945545	69	13,86
20	-3,334024	70	14,23
22	-2,779396	61	13,13
24	-3,113298	65	12,59
23	-2,981064	48	14,12
20	-2,680835	46	14,83
18	-2,999039	55	14,3
18	-2,586609	44	15,05
22	-2,785642	52	12,83
21	-3,015559	54	12,03
20	-2,703407	48	16
14	-2,890193	48	13,68
15	-3,016932	48	15,7
18	-2,748745	44	14,81
25	-2,64936	46	15,48
21	-2,800492	61	12,47
19	-2,902526	54	13,77
18	-3,182431	58	17,24
23	-3,377713	59	14,76
17	-3,094103	48	14,04
17	-2,511458	55	15,39
20	-3,030631	56	13,23
22	-2,861027	43	15,54
19	-2,934551	42	15,61

FMS fundamental motor skills.
